# Causes of long-term mortality in patients with head and neck squamous cell carcinomas

**DOI:** 10.1007/s00405-021-07211-8

**Published:** 2021-12-14

**Authors:** Joan Lop, María del Prado Venegas, Albert Pujol, Blanca Sauter, Rosselin Vásquez, María Casasayas, Miquel Quer, Xavier León

**Affiliations:** 1 Pathology Department, Hospital del Mar, Universitat Autònoma de Barcelona, Barcelona, Spain; 2 Otorhinolaryngology Department, Hospital de la Santa Creu i Sant Pau, Universitat Autònoma de Barcelona, C/ Mas Casanovas, 90, 08041 Barcelona, Spain; 3 Centro de Investigación Biomédica en Red de Bioingeniería, Biomateriales y Nanomedicina (CIBER-BBN), Madrid, Spain

**Keywords:** Head and neck cancer, Survival, Causes of death, Competing mortality, Long-term mortality

## Abstract

**Purpose:**

After treatment of a head and neck squamous cell carcinoma (HNSCC), patients with an adequate control of the tumor have a decreased overall survival when compared to age- and gender-matched controls in the general population. The aim of our study was to analyze the causes of long-term mortality in patients with HNSCC.

**Methods:**

We carried out a retrospective study of 5122 patients with an index HNSCC treated at our center between 1985 and 2018. We analyzed the survival considering three causes of death: mortality associated with the HNSCC index tumor, mortality associated with a second or successive neoplasm, and mortality associated with a non-cancer cause.

**Results:**

After the diagnosis of an HNSCC the most frequent cause of death is the head and neck tumor itself during the first 3.5 years of follow-up. Thereafter, mortality is more frequently associated with competing causes of death, such as second malignancies and non-cancer causes. Mortality associated with second and successive neoplasms was 2.3% per year, a percentage that was maintained constant throughout the follow-up. Likewise, mortality attributable to non-cancer causes was 1.6% per year, which also remained constant. There were differences in the mortality patterns according to the characteristics of the patients.

**Conclusion:**

There are differences in the mortality patterns of patients with HNSCC depending on their characteristics. Knowledge of these patterns can help in the design of guidelines to improve the follow-up protocols of this group of patients to optimize the clinical cost-effectiveness.

**Supplementary Information:**

The online version contains supplementary material available at 10.1007/s00405-021-07211-8.

## Introduction

After diagnosis and treatment of head and neck squamous cell carcinomas (HNSCC), patients who achieve an adequate control and cure of the tumor have a decreased overall survival in the medium to long term when compared to age- and gender-matched controls in the general population [1]. In a study carried out from a register of oncologic patients treated in the USA, Massa et al. [2] found that life expectancies for those diagnosed with HNSCC were 6.5 years lower than estimated once mortality associated with the head and neck tumor was excluded.

Similarly, van der Schroeff et al. [3] described that surviving patients after HNSCC had between 20 and 25 percent increased risk of death compared to the general population. These differences in survival are largely due to the increased risk of second and other successive malignancies, the burden of comorbidities associated with tobacco and alcohol use in this type of patients, and the morbidity associated with the oncologic treatments used [4–6].

There is limited information on long-term survival and causes of death in patients with HNSCC. Some studies, mostly based on population-based registries, provide information on overall or disease-specific survival, but do not detail the causes of mortality [7–9], or have a limited follow-up period [1, 5].

The knowledge of the timing and causes of death of patients with a HNSC according to their characteristics may help in the design of guidelines to optimize the clinical cost-effectiveness of follow-up protocols for this group of patients.

The aim of our study was to analyze the causes of long-term mortality in patients with HNSCC taking into consideration variables, such as age and gender, history of tobacco and alcohol abuse, location and extent of the tumor, and the type of treatment.

## Materials and methods

The present study was performed retrospectively using a database that prospectively collects information regarding the clinical and oncologic characteristics, treatment, and follow-up of all patients with malignant head and neck tumors treated at our center since 1985 [10].

The criteria that had to be met by the patients included in this study were: a positive biopsy for squamous cell carcinoma of the head and neck; having had treatment in our center between 1985 and 2018; and a follow-up of at least 24 months. In case the patient had had more than one HNSCC, only the initial index tumor was considered, including tumors diagnosed subsequently as second or successive neoplasm.

In the definition of a second or any successive neoplasms we followed the criteria established by Warren and Gates [11]: to have histologic confirmation of each second primary neoplasm, to rule out the possibility that one of the tumors is a metastasis or recurrence of the previous tumor, and in tumors of adjacent locations, to rule out the existence of a submucosal connection between both tumors.

During the mentioned study period, a total population of 5214 patients with an index HNSCC were treated at our center. Ninety-two patients with a follow-up of less than 24 months after the end of treatment were excluded. Consequently, a significant cohort of 5122 patients met the criteria. The mean follow-up period of the patients was 6.5 years (standard deviation 6.0 years).

Table 1 shows the characteristics of the patients included in the study. Given the interaction between tobacco and alcohol use, a combined variable of tobacco and alcohol abuse was created with three categories: no tobacco or alcohol abuse; moderate tobacco and/or alcohol abuse (< 20 cigarettes/day and/or < 80 g alcohol/day); and severe tobacco and/or alcohol abuse (≥ 20 cigarettes/day or ≥ 80 g alcohol/day). All patients were evaluated by an oncologic committee that carried out the tumor TNM classification and proposed a treatment according to the institutional clinical guidelines. Staging was performed according to the current TNM edition at the time of diagnosis. One hundred and nineteen patients with carcinomas in situ and 124 patients with cervical lymph node metastases with unknown primary were grouped into the local cT1 extension category. Treatments performed on the primary tumor location were grouped in three categories: palliative treatment (including supportive treatment and palliative chemotherapy), surgical treatment (including patients treated with adjuvant radiotherapy or chemoradiotherapy), and radiotherapy (including patients treated with chemoradiotherapy or bioradiotherapy). For patients with cervical lymph node metastasis with unknown primary, we took into consideration the treatment carried out on the lymph node areas. Table 1 of the Supplementary Material includes information on the type of treatment carried out according to the primary location of the tumor.

**Table 1:** Characteristics of the patients included in the study

	*N*	%
Gender		
Male	4554	88.9
Female	568	11.1
Age		
< 50 years	810	15.8
50–70 years	3067	59.9
> 70 years	1245	24.3
Tobacco		
No	586	11.4
< 20 cigarettes/day	796	15.5
≥ 20 cigarettes/day	3743	73.1
Alcohol		
No	1235	24.1
< 80 g alcohol/day	2082	40.6
≥ 80 g alcohol/day	1805	35.2
Tobacco and alcohol abuse		
No	477	9.3
Moderate	781	15.2
Severe	3864	75.4
Stage		
I	1289	25.2
II	823	16.1
III	1143	22.3
IV	1867	36.5
Location		
Oral cavity	656	12.8
Oropharynx	950	18.5
Hypopharynx	437	8.5
Larynx	2599	50.7
Rhinopharynx	243	4.7
Unknown primary	124	2.4
Nasal cavities and sinuses	113	2.2
Treatment		
Palliative	285	5.6
Surgery	1666	32.5
Surgery	741	14.5
Surgery + (chemo)radiotherapy	925	18.1
Radiotherapy	3171	61.9
Radiotherapy	2573	50.2
Chemoradiotherapy	598	11.7

Information regarding the human papillomavirus (HPV) status was available for 372 of the patients with an oropharyngeal carcinoma diagnosed since 1991. Human papilloma virus (HPV) DNA detection and genotyping in patients with an oropharyngeal carcinoma was evaluated retrospectively with the SPF-10 PCR/DEIA/ LiPA25 system up to 2012, and with the PCR/CLART.

HPV2 system by GENOMICA (GENOMICA S.A.U., Madrid, Spain) prospectively thereafter. Immunohistochemical positivity against p16INKa was evaluated for all positive PCR samples. The requirement to consider a p16INKa positive sample was staining of at least 70% of the tumor cells at the nuclear or cytoplasmic level with a moderate or high staining intensity. HPV-positive oropharyngeal carcinomas were those with the presence of viral DNA and immunohistochemical positivity to p16INKa. A total of 75 patients (20.2%) had HPV-positive tumors.

We carried out an analysis of the survival from the date of diagnosis of the HNSCC index tumor. Three causes of mortality were considered in this analysis: mortality associated with the HNSCC index tumor, mortality associated with a second or successive neoplasm, and mortality associated with a non-cancer cause. The mortality analysis was performed considering the overall number of patients, as well as according to relevant variables, such as gender, patient age, tobacco and alcohol abuse, tumor staging (early stages I–II vs advanced stages III–IV), the main locations of the primary tumor, and the type of treatment performed. For the patients with oropharyngeal carcinomas, a survival analysis was performed according to the HPV status.

We analyzed the conditional survival of surviving an additional 3 months during the first 5 years of follow-up considering either the mortality attributable to the HNSCC index tumor, or the competitive causes of death, including the mortality associated with second or successive neoplasms and that due to non-cancerous causes.

The estimation of survival was carried out with the Kaplan–Meier method, using the log-rank test in the comparison of survival curves. Conditional survival was calculated according to the formula proposed by Hieke et al. [12].

All procedures were reviewed by the Institutional Review Board of our center. The study was conducted in accordance with the principles outlined in the Declaration of Helsinki. Because of the retrospective nature of the study, no formal consent was required.

## Results

Five-, 15- and 25-year overall survival of the patients included in the study was 56.8%, 33.5% and 16.7%, respectively. Figure 1 in the Supplementary Material shows the overall survival at 25 years. During the follow-up period a total of 1,556 patients (30.4%) died as a consequence of the HNSCC index tumor, 906 (17.7%) as a consequence of a second or successive neoplasm (of which 225 patients (4.4%), from a second neoplasm located again in the head and neck), and 598 (11.7%) from a non-cancer cause. Table 2 shows the survival values at 5, 10, 15, 20, and 25 years after diagnosis of the HNSCC index tumor according to the different causes of death.

**Table 2:** Survival according to the different causes of mortality

Cause of mortality	5-year surv (CI 95%)	10-year surv (CI 95%)	15-year surv (CI 95%)	20-year surv (CI 95%)	25-year surv (CI 95%)
HNSCC	69.3% (67.9–70.7)	66.7% (65.3–68.1)	66.3% (64.9–67.7)	66.3% (64.9–67.7)	66.3% (64.9–67.7)
2nd neoplasm	89.7% (88.7–90.7)	76.7% (75.1–78.3)	65.7% (63.3–68.1)	51.9% (48.4–55.4)	42.3% (37.8–46.8)
Noncancer	91.4% (90.4–92.4)	84.4% (83.0–85.8)	77.1% (74.9–79.3)	68.9% (65.6–72.2)	60.2% (55.1–65.3)

**Fig. 1:**
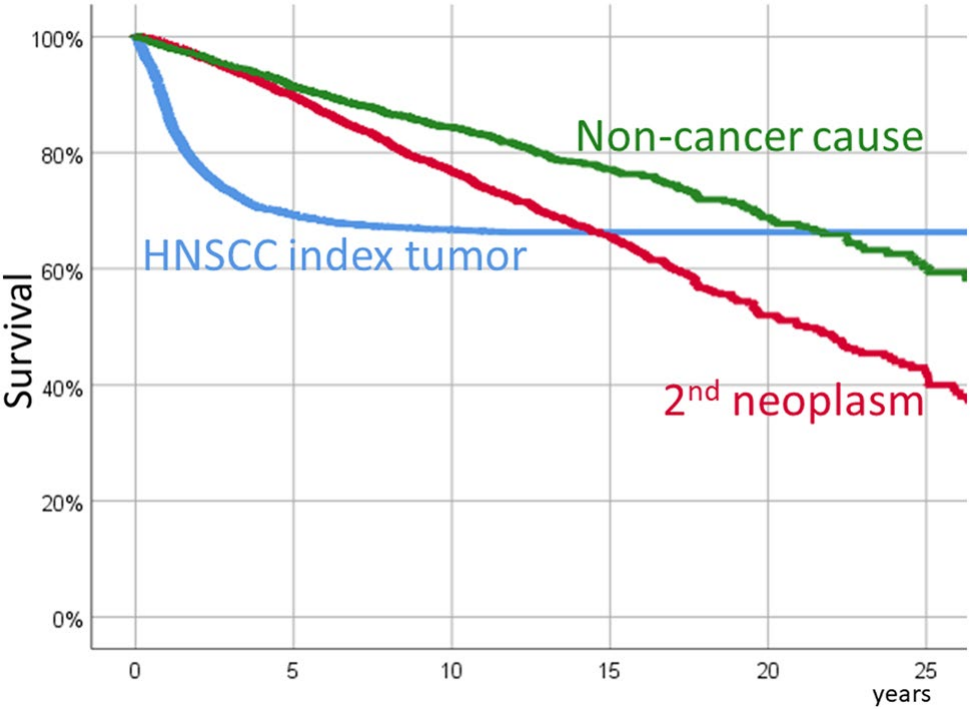
Long-term survival according to the different causes of mortality

Figure 1 shows the survival curves depending on the cause of death. The mortality attributable to the HNSCC index tumor mostly occurred during the first 5 years of follow-up (71.6% of deaths due to the index tumor occurred during the first 2 years of follow-up). Mortality associated with second and successive neoplasms was 2.3% per year, a percentage that held near constant throughout the follow-up. Likewise, mortality attributable to non-cancer causes was 1.6% per year, which also remained constant.

Table 3 shows the survival values according to the different causes of death at 5, 10, 15, 20 and 25 years of follow-up correlated to variables, such as gender, age of the patient at the time of diagnosis of the index tumor, tobacco and/or alcohol abuse, staging, main locations of the primary tumor, and whether the patient was treated with surgery or radiotherapy.

**Table 3:** Survival according to the different causes of mortality depending on different variables

			5-year surv	10-year surv	15-year surv	20-year surv	25-year surv	*P*
Gender	HNSCC	Male	69.6%	67.1%	66.8%	66.8%	66.8%	0.018
		Female	66.7%	63.2%	62.3%	62.3%	62.3%	
	2nd neoplasm	Male	89.1%	75.3%	63.7%	49.0%	41.1%	0.0001
		Female	95.3%	88.9%	82.0%	76.0%	54.3%	
	Noncancer	Male	90.9%	83.6%	75.8%	67.3%	58.0%	0.0001
		Female	96.2%	91.2%	87.7%	80.9%	75.9%	
Age	HNSCC	< 50 years	68.6%	66.1%	65.8%	65.8%	65.8%	0.004
		50–70 years	70.5%	68.1%	67.5%	67.5%	67.5%	
		> 70 years	66.8%	63.6%	63.6%	63.6%	63.6%	
	2nd neoplasm	< 50 years	93.6%	82.5%	73.0%	57.4%	51.0%	0.0001
		50–70 years	89.0%	76.4%	65.4%	52.0%	40.3%	
		> 70 years	88.9%	71.4%	55.3%	33.2%	0.0%	
	Noncancer	< 50 years	97.8%	95.9%	92.8%	88.3%	80.5%	0.0001
		50–70 years	93.9%	88.9%	81.5%	70.6%	60.2%	
		> 70 years	79.9%	60.8%	43.4%	26.0%	26.0%	
Tobacco and alcohol abuse	HNSCC	No	68.7%	64.7%	63.6%	63.6%	63.6%	0.007
		Moderate	74.7%	71.2%	70.3%	70.3%	70.3%	
		Severe	68.2%	66.0%	65.8%	65.8%	65.8%	
	2nd neoplasm	No	96.8%	92.1%	88.7%	78.8%	62.1%	0.0001
		Moderate	92.1%	81.2%	73.5%	60.5%	49.9%	
		Severe	88.3%	73.9%	61.4%	47.1%	37.9%	
	Noncancer	No	94.6%	87.4%	84.4%	74.0%	67.0%	0.029
		Moderate	92.1%	84.6%	78.7%	72.8%	70.2%	
		Severe	90.9%	84.0%	75.8%	67.5%	56.2%	
Stage	HNSCC	I–II	90.8%	88.8%	88.6%	88.6%	88.6%	0.0001
		III–IV	53.9%	50.9%	50.3%	50.3%	50.3%	
	2nd neoplasm	I–II	91.9%	80.6%	70.0%	57.7%	47.0%	0.0001
		III–IV	87.2%	72.1%	60.8%	45.6%	37.3%	
	Noncancer	I–II	92.6%	85.6%	77.6%	69.2%	59.1%	0.064
		III–IV	90.4%	83.2%	76.8%	69.6%	62.2%	
Location	HNSCC	Oral cavity	63.1%	60.6%	60.1%	60.1%	60.1%	0.0001
		Oropharynx	50.9%	48.1%	48.1%	48.1%	48.1%	
		Hypopharynx	49.0%	47.8%	47.0%	47.0%	47.0%	
		Larynx	82.9%	81.1%	81.0%	81.0%	81.0%	
	2nd neoplasm	Oral cavity	90.1%	77.7%	64.9%	54.3%	35.7%	0.0001
		Oropharynx	86.7%	66.6%	49.1%	29.9%	22.9%	
		Hypopharynx	85.5%	60.7%	53.0%	33.3%	23.8%	
		Larynx	90.0%	79.1%	68.7%	55.4%	46.6%	
	Noncancer	Oral cavity	92.8%	85.5%	77.5%	71.2%	52.2%	0.538
		Oropharynx	90.6%	82.9%	77.0%	67.4%	62.2%	
		Hypopharynx	88.6%	81.5%	76.0%	65.2%	65.2%	
		Larynx	91.6%	84.3%	76.4%	68.3%	59.5%	
Treatment	HNSCC	Surgery	74.9%	73.1%	72.7%	72.7%	72.7%	0.007
		Radiotherapy	72.1%	69.0%	68.4%	68.4%	68.4%	
	2nd neoplasm	Surgery	89.7%	78.5%	68.2%	53.1%	42.7%	0.461
		Radiotherapy	90.1%	76.1%	64.6%	51.6%	42.6%	
	Noncancer	Surgery	90.4%	84.0%	76.4%	67.7%	61.2%	0.289
		Radiotherapy	92.1%	84.6%	77.5%	69.5%	59.2%	
HPV status*	HNSCC	HPV negative	53.8%	51.9%	51.9%	51.9%	51.9%	0.001
		HPV positive	74.8%	72.1%	72.1%	72.1%	72.1%	
	2nd neoplasm	HPV negative	80.8%	55.3%	31.0%	13.8%	9.2%	0.002
		HPV positive	92.2%	81.0%	81.0%	60.8%	30.4%	
	Noncancer	HPV negative	88.2%	78.7%	70.6%	70.6%	70.6%	0.310
		HPV positive	94.5%	84.7%	84.7%	84.7%	84.7%	

*372 patients with oropharyngeal carcinoma

Survival related to the HNSCC index tumor was significantly lower in the case of female patients, with advanced age, in patients with no history of tobacco or alcohol abuse, with an advanced-stage tumor, with a primary tumor located in the oro-hypopharynx, and in those who received treatment with radiotherapy.

For mortality associated with second and subsequent neoplasms, the worst survival was found in male patients, of advanced age, with high tobacco and/or alcohol abuse, with an advanced-stage tumor, or with tumors located in the oro-hypopharynx. Finally, mortality from non-cancer causes was higher in male patients, with advanced age, and in those with a history of severe tobacco and/or alcohol abuse. Neither the extension of the tumor, nor its location, or the type of treatment used were significantly related to the risk of death not associated with oncologic causes.

For oropharyngeal carcinomas, patients with HPV-negative tumors had a significant reduction in survival related to the index tumor and second malignancies, while no differences appeared in relation to deaths unrelated to oncologic pathology.

**Fig. 2:**
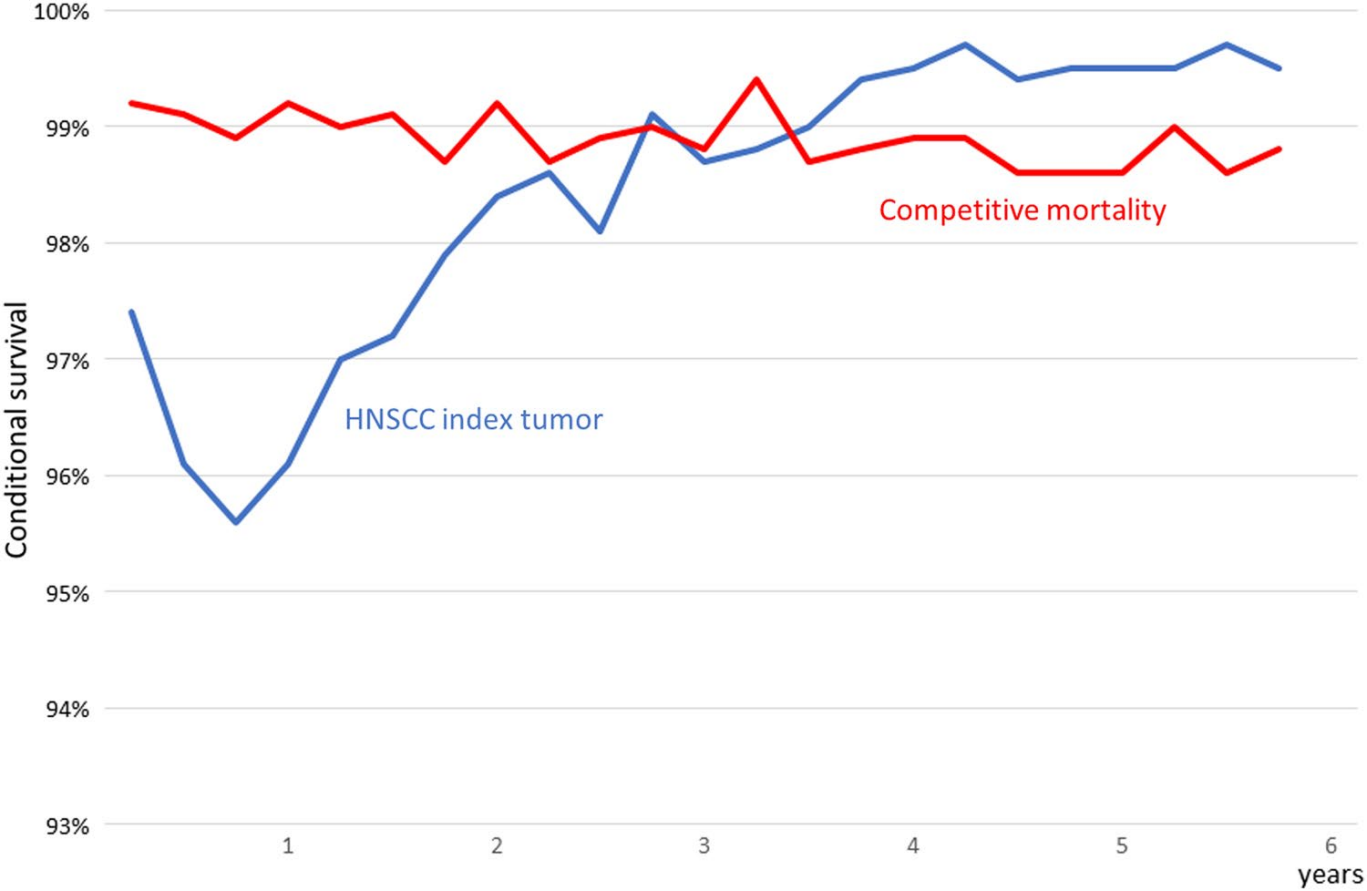
Conditional survival during the first 5 years of follow-up depending on whether the patient died as a result of the HNSCC index tumor or of competing causes

The Supplementary Material shows the survival curves according to the causes of death as a function of gender, age, tobacco and alcohol abuse, tumor extension, location of the primary tumor and treatment used (Figs. 2–7 of the Supplementary Material). For patients with oropharyngeal carcinomas, we determined the survival according to the different causes of death as a function of HPV status (Fig. 8 of the Supplementary Material).

Table 2 of the Supplementary Material shows the survival values according to the different causes of death as a function of the separate uses of tobacco and alcohol, and of the treatment of the index tumor considering the use of adjuvant treatment with postoperative radiotherapy, or treatment with chemoradiotherapy vs radiotherapy alone.

Figure 2 shows the conditional survival related to mortality attributable to the HNSCC index tumor and to the competing causes of death. The conditional survival related to HNSCC was lower during the first 3 years of follow-up, while that related to competing causes of death remained practically stable. After 42 months of follow-up, mortality related to competing causes of death became more common than that associated with HNSCC index tumor.

## Discussion

According to our results, after 3.5 years of follow-up, patients with HNSCC were more likely to die as a consequence of a cause other than the HNSCC index tumor. The most frequent cause of death after 3.5 years of follow-up was a new malignant tumor, which resulted in an annual death rate of 2.3% per year, and which remained constant throughout the entire follow-up period since the diagnosis of HNSCC. In addition, there was a non-cancer related mortality rate of 1.6% per year, which also remained constant throughout the entire follow-up period.

Few studies have analyzed the causes of long-term mortality in patients with HNSCC. Baxi et al. [5] evaluated the mortality patterns of patients with HNSCC included in the SEER (Surveillance, Epidemiology, and End Results), a population-based registry of oncologic patients treated in the USA. They analyzed the 10-year follow-up of a cohort of 35,958 patients who had survived a minimum of 3 years after the diagnosis of a HNSCC. As in our study, the authors described a mortality associated with the appearance of a second neoplasm and from non-cancer causes that remained constant throughout the follow-up period, with annual mortality rates of 1.1% associated with a second neoplasm and 1.8% per year from non-cancer causes. In this study, the risk of death associated with the appearance of a second neoplasm was higher for patients older than 60 years at the time of diagnosis of the HNSCC index tumor, in patients with advanced tumors, in those with hypopharyngeal location, and in those treated with radiotherapy.

Similarly, in a study analyzing the competing causes of mortality at 10 years in 34,568 patients with HNSCC included in the SEER registry, Rose et al. [13] reported that HNSCC-associated mortality predominantly occurred during the first 3 years after treatment, and that mortality from second malignancies and non-cancer causes remained constant throughout the follow-up period.

Finally, in another study of 151,155 patients with HNSCC from the SEER registry, Simpson et al. [14] found that death from competing causes became more common than death from HNSCC index tumor after 4.75 years of follow-up.

Tiwana et al. [7] analyzed the long-term survival of a cohort of 1,657 patients with HNSCC diagnosed in a Canadian province during the period 1986–1990, and found an overall survival at 5, 15 and 25 years of 46%, 21% and 11%, and a disease-specific survival of 63%, 53% and 49%, respectively. Male patients, older at diagnosis, with advanced tumor and with tumors located in the oro-hypopharynx had the worst overall and disease-specific survival.

The most frequent cause of competitive mortality after the 3rd year of follow-up for our patients was related to the appearance of second and successive neoplasms. In a previous study we were able to appreciate how after the diagnosis of a HNSCC, patients had an incidence of second neoplasm of 3.5% per year, which remained practically constant over a follow-up period of 30 years [15]. In addition, patients who had a second neoplasm had an even higher risk of having a third neoplasm, and patients with a third neoplasm had a higher risk of having a fourth tumor. Most of these second and successive neoplasms appeared at the level of the aerodigestive tract, including locations with limited disease-specific survival, such as lung and esophageal carcinomas. This pattern in the appearance of new tumors justifies the high mortality related to new neoplasms, which we were able to quantify at 2.3% per year, and which remained constant throughout the follow-up. Mortality associated with second and successive neoplasms was significantly more frequent in patients of male gender, of advanced age, with a high tobacco and/or alcohol abuse, with advanced HNSCC, and with tumors located in the oro-hypopharynx.

The percentage of non-cancer mortality also remained constant throughout the follow-up period, and we quantified it at 1.6% per year. As expected, non-cancer mortality increased progressively with increasing patient age at the time of diagnosis of the HNSCC index tumor. The other variables that were significantly related to an increase in non-cancer mortality were male gender and high tobacco and/or alcohol abuse. This increase in mortality of non-cancer origin can be attributed to the disproportionate burden of morbidities associated with the high abuse of toxic substances [4, 5], which in our country appears preferentially in male patients. Unfortunately, our database does not include information on the cause of death when it does not have an oncologic etiology. In the study carried out by Rose et al. [13] on patients included in the SEER registry, the most frequent causes of non-cancer mortality in patients with a HNSCC were cardiovascular diseases, chronic respiratory pathologies and cerebrovascular accidents. For patients with oropharyngeal carcinomas, differences in long-term survival patterns have been described depending on the HPV status. In a study analyzing the competitive mortality of patients with oropharyngeal carcinoma, Lop et al. [16] found that patients with HPV-positive tumors and no history of tobacco or alcohol abuse, in addition to having a lower mortality associated with the oropharyngeal tumor, had a significantly lower mortality associated with the appearance of second neoplasms, and a significantly lower non-cancer-related mortality than HPV-negative patients.

One of the strengths of our study is that it analyses a cohort of consecutively recruited patients with a long follow-up time. In addition, the information was collected in a standardized and prospective manner, which assures the quality and uniformity of the data included in the study. However, the present study has a series of limitations related to its retrospective nature. Knowledge of the comorbidities existing at the time of diagnosis of the HNSCC was very limited, so it could not be included as a variable. We did not have information on the final cause of death for patients who did not die because of a tumor. Likewise, we did not have information on the persistence of tobacco and alcohol consumption after the diagnosis and treatment of the HNSCC index tumor, a variable that could be related to the risk of competitive mortality. On the other hand, this is a single-center study including a patient population with a high burden in the abuse of toxic substances, such as tobacco and alcohol, and with a high proportion of patients with tumors located in the larynx. We consider that the epidemiological characteristics of our population are an element to be considered when analyzing the results and comparing them with those obtained in other studies.

The evidence presented in our study shows that the most common cause of death after diagnosis of a HNSCC index tumor is the tumor itself during the first 3.5 years of follow-up. Consequently, during this period the oncological controls should be focused on the early detection of tumor recurrence to offer, when possible, salvage treatment. On the other hand, after the first 3.5 years of follow-up patients with HNSCC face a considerable risk of death not associated with the head and neck index tumor, including a mortality rate due to second malignancies and non-cancer causes higher than that of the general population. To improve overall survival, long-term follow-up protocols for these patients should be designed with consideration of screening for the most prevalent second malignancies, treatment of comorbidities, and modification of known risk factors, such as tobacco and alcohol abuse.

## Conclusions

After the diagnosis of a HNSCC the most frequent cause of death is the head and neck tumor itself during the first 3.5 years of follow-up. Thereafter, mortality is more frequently associated with competing causes of death, such as second malignancies and non-cancer causes. Mortality associated with second malignancies was 2.3% per year and that related to non-cancer causes was 1.6% per year, both remaining constant throughout the follow-up period. There were significant differences in the pattern of causes of death according to variables, such as age, gender, tobacco and alcohol abuse, and the extension and location of the HNSCC index tumor.

## Supplementary Information

Below is the link to the electronic supplementary material.

Supplementary file1 (DOCX 872 KB)
