# Supplementary material for: Causes of long-term mortality in patients with head and neck squamous cell carcinomas
Source: Eur Arch Otorhinolaryngol. 2021 Dec 14;279(7):3657–64. doi: 10.1007/s00405-021-07211-8 (PMC9130168; doi:10.1007/s00405-021-07211-8)
Supplement: Supplementary file 1 — Supplementary file1 (DOCX 872 KB) [file 405_2021_7211_MOESM1_ESM.docx]

**Supplementary material.**

**Figure 1.** Long-term overall survival of the patients included in the study.


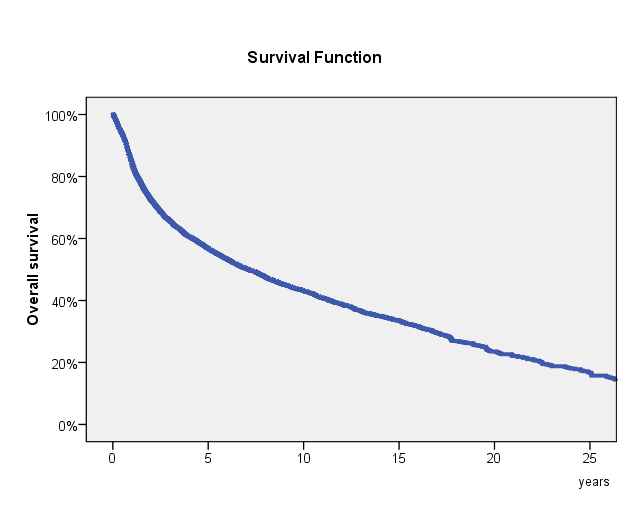


**Figure 2.** Long-term survival according to the different causes of mortality depending on the gender.


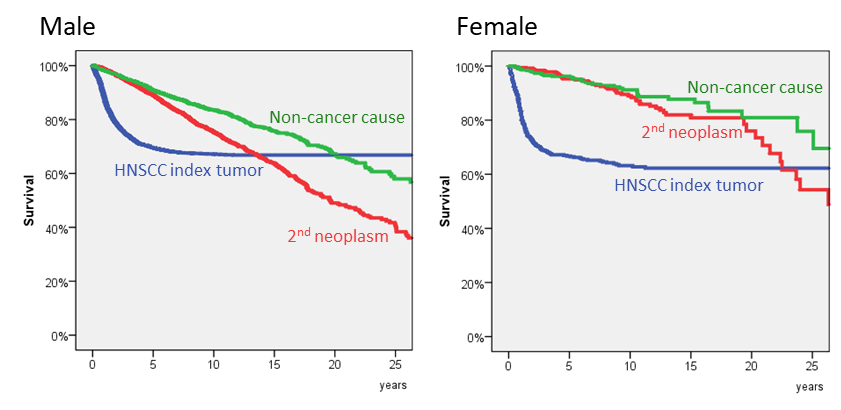


**Figure 3.** Long-term survival according to the different causes of mortality depending on the patient’s age at the time of diagnosis of the HNSCC index tumor.


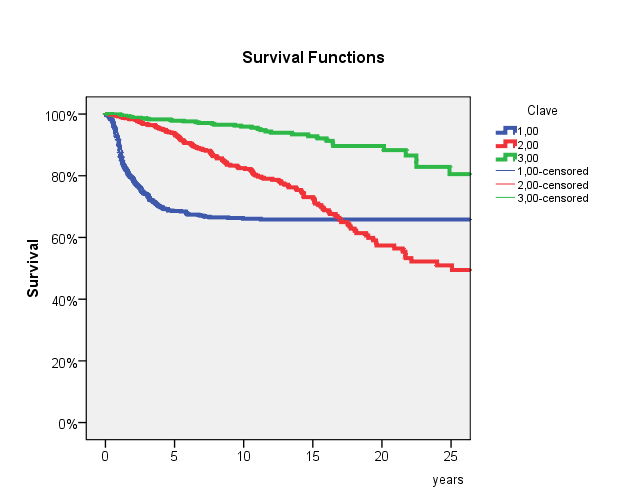

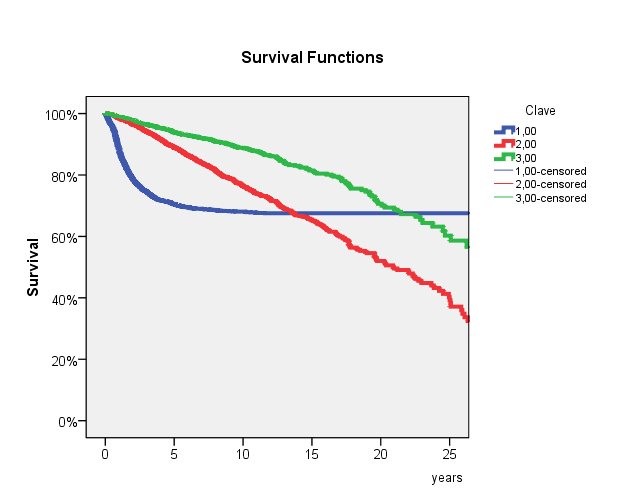


Non-cancer cause

2^nd^ neoplasm

HNSCC index tumor

Non-cancer cause

2^nd^ neoplasm

< 50 years

50-70 years


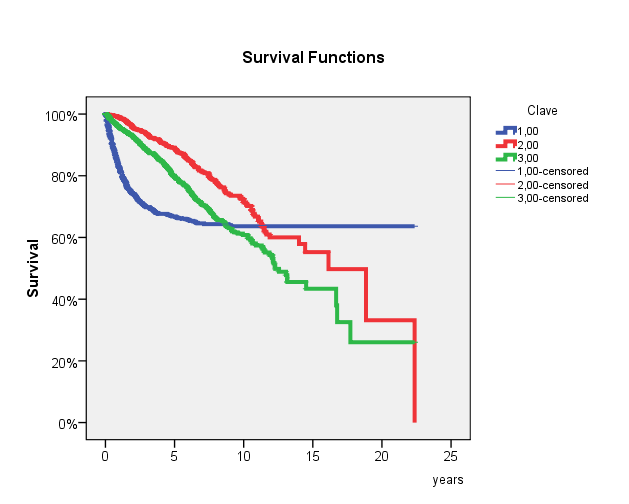


> 70 years

HNSCC index tumor

Non-cancer cause

**Figure 4.** Long-term survival according to the different causes of mortality depending on the tobacco and alcohol abuse.


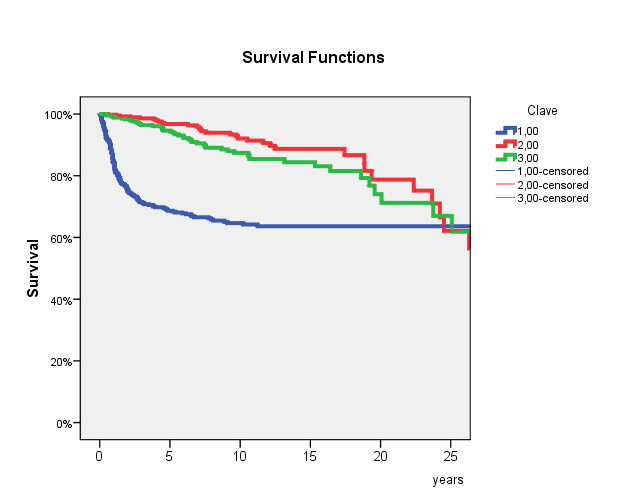

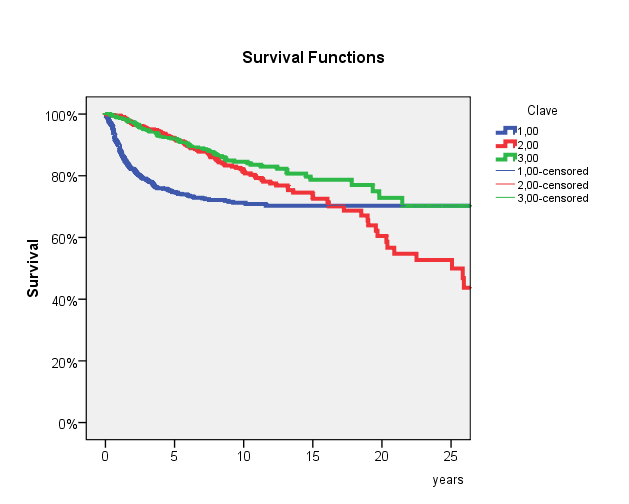

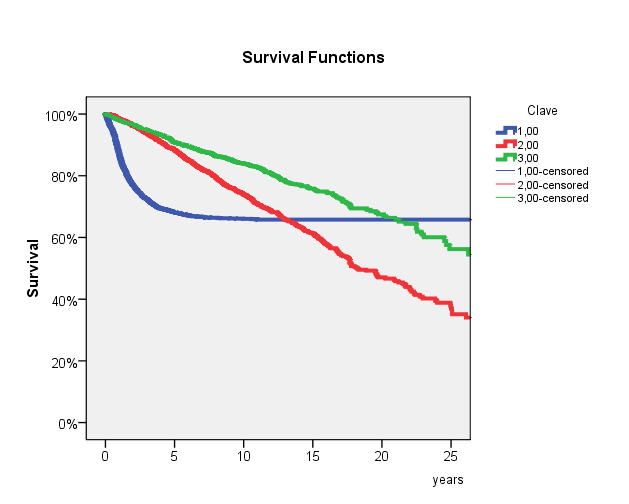


HNSCC index tumor

Non-cancer cause

2^nd^ neoplasm

HNSCC index tumor

Non-cancer cause

2^nd^ neoplasm

No tobacco or alcohol

Moderate abuse

HNSCC index tumor

Non-cancer cause

2^nd^ neoplasm

Severe abuse

**Figure 5.** Long-term survival according to the different causes of mortality depending on the tumor extension.


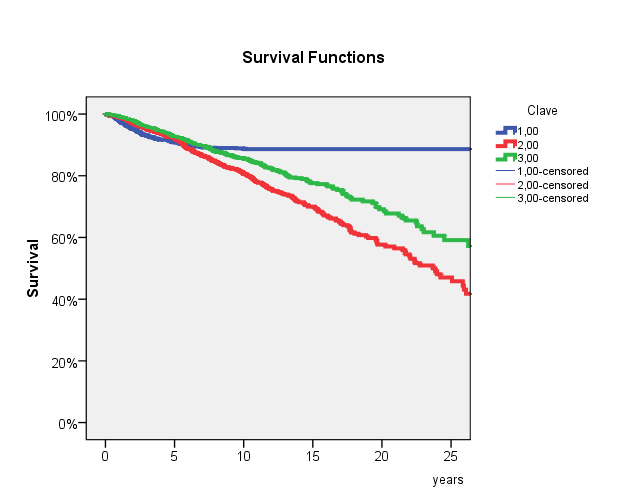

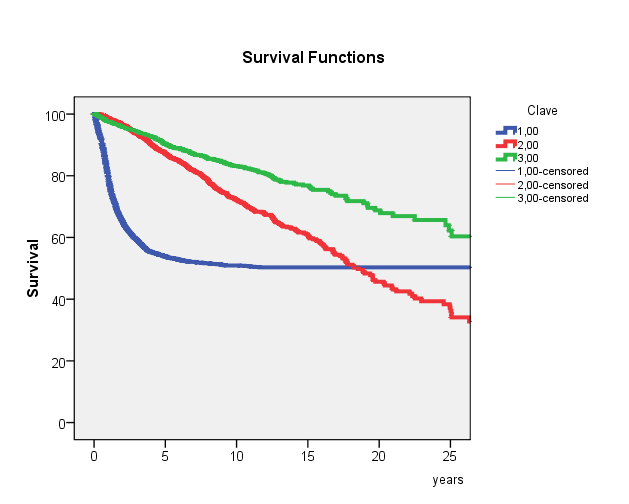


HNSCC index tumor

Non-cancer cause

2^nd^ neoplasm

HNSCC index tumor

Non-cancer cause

2^nd^ neoplasm

Stage I-II

Stage III-IV

**Figure 6.** Long-term survival according to the different causes of mortality depending on the location of the primary tumor.


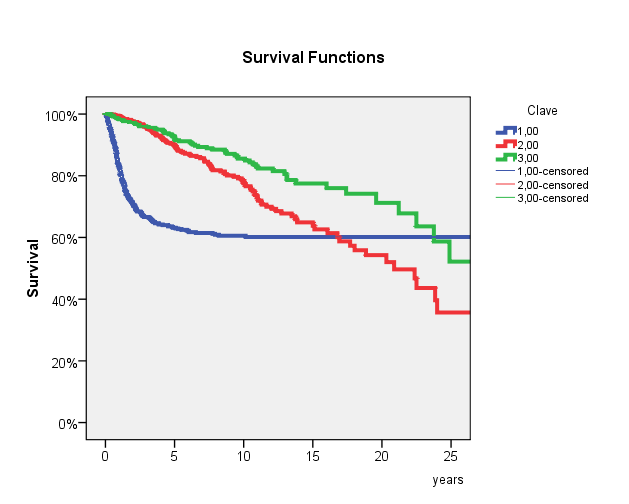

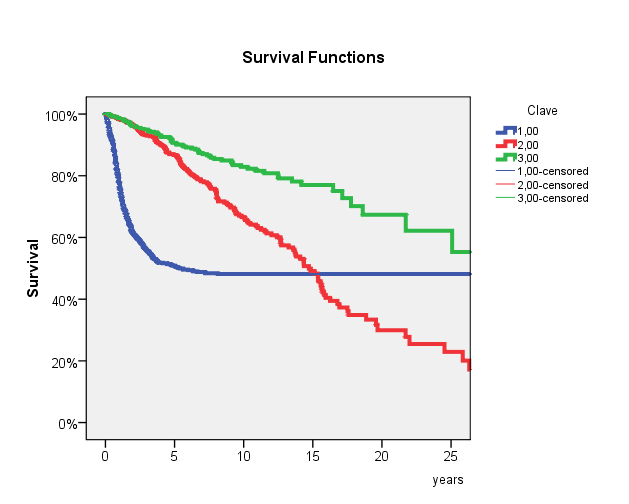

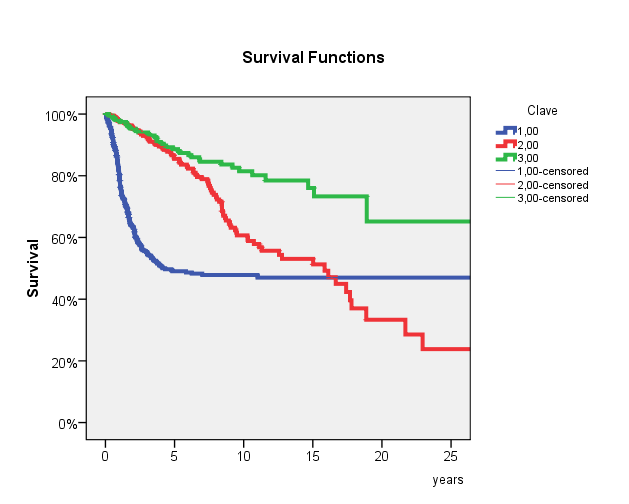

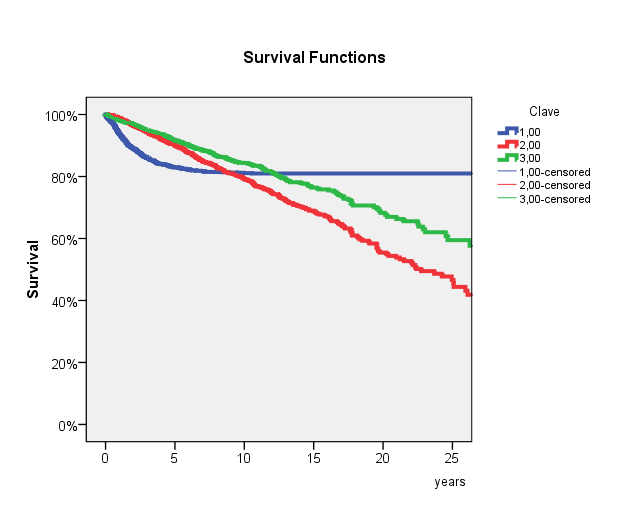


Non-cancer cause

Non-cancer cause

Oral cavity

Oropharynx

2^nd^ neoplasm

2^nd^ neoplasm

HNSCC index tumor

Non-cancer cause

2^nd^ neoplasm

HNSCC index tumor

Non-cancer

cause

2^nd^ neoplasm

HNSCC index tumor

Hypopharynx

Larynx

**Figure 7.** Long-term survival according to the different causes of mortality depending on the treatment used.


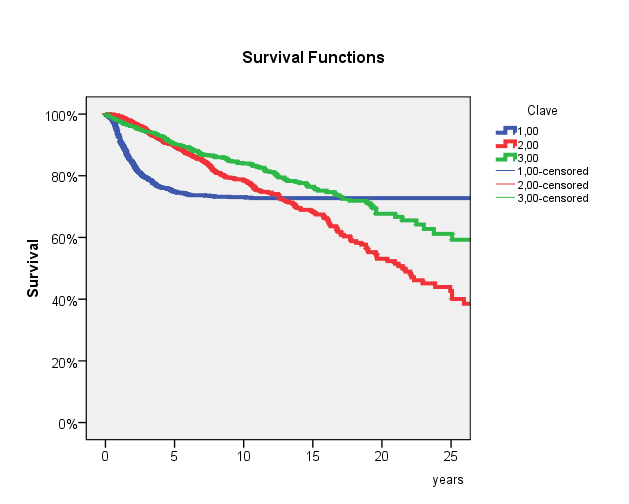

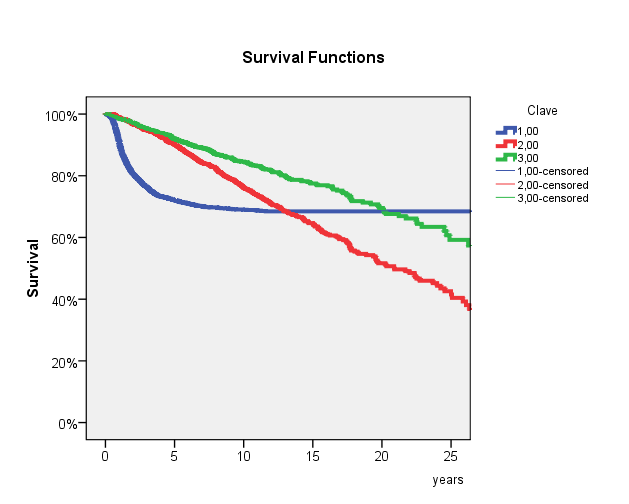


HNSCC index tumor

Non-cancer cause

Non-cancer cause

Surgery

Radiotherapy

2^nd^ neoplasm

2^nd^ neoplasm

HNSCC index tumor

**Figure 8.** Long-term survival according to the different causes of mortality depending on the HPV status for patients with oropharyngeal carcinoma.

**
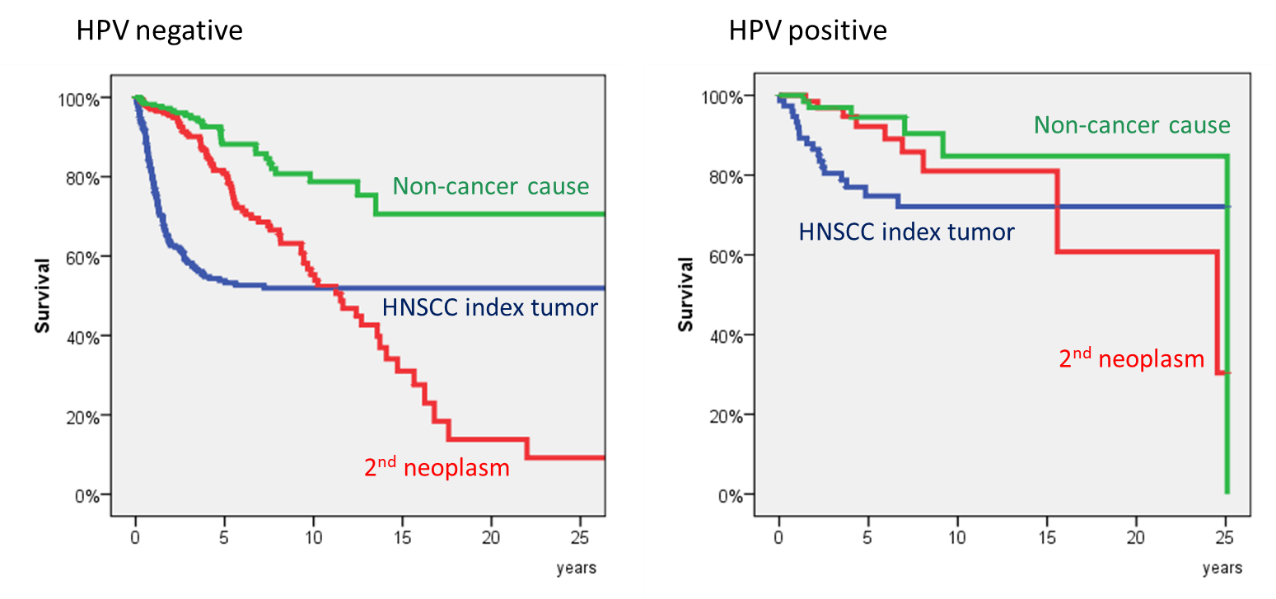
**

**Table 1.** Type of treatment carried out according to the primary location of the tumor.

| **Location** | **Treatment** | **N** | **%** |
| --- | --- | --- | --- |
| Oral cavity | Palliative | 42 | 6.4 |
|  | Surgery | 197 | 30.0 |
|  | Surgery + (chemo)radiotherapy | 165 | 25.2 |
|  | Radiotherapy | 224 | 34.1 |
|  | Chemoradiotherapy | 28 | 4.3 |
| Oropharynx | Palliative | 99 | 10.4 |
|  | Surgery | 39 | 4.1 |
|  | Surgery + (chemo)radiotherapy | 69 | 7.3 |
|  | Radiotherapy | 494 | 52.0 |
|  | Chemoradiotherapy | 249 | 26.2 |
| Hipopharynx | Palliative | 37 | 8.5 |
|  | Surgery | 17 | 3.9 |
|  | Surgery + (chemo)radiotherapy | 111 | 25.4 |
|  | Radiotherapy | 180 | 41.2 |
|  | Chemoradiotherapy | 92 | 21.1 |
| Larynx | Palliative | 81 | 3.1 |
|  | Surgery | 459 | 17.7 |
|  | Surgery + (chemo)radiotherapy | 468 | 18.0 |
|  | Radiotherapy | 1,458 | 56.1 |
|  | Chemoradiotherapy | 133 | 5.1 |
| Rhinopharynx | Palliative | 6 | 2.5 |
|  | Surgery | - |  |
|  | Surgery + (chemo)radiotherapy | - |  |
|  | Radiotherapy | 158 | 65.0 |
|  | Chemoradiotherapy | 79 | 32.5 |
| Unknown primary | Palliative | 12 | 9.7 |
|  | Surgery | 18 | 14.5 |
|  | Surgery + (chemo)radiotherapy | 80 | 64.5 |
|  | Radiotherapy | 12 | 9.7 |
|  | Chemoradiotherapy | 2 | 1.6 |
| Nasal cavities and sinuses | Palliative | 8 | 7.1 |
|  | Surgery | 11 | 9.7 |
|  | Surgery + (chemo)radiotherapy | 32 | 28.3 |
|  | Radiotherapy | 47 | 41.6 |
|  | Chemoradiotherapy | 15 | 13.3 |

**Table 2.** Survival according to the different causes of mortality depending on the use of tobacco and alcohol and the treatment carried out on the primary tumor site.

|  |  |  | **5-year surv** | **10-year surv** | **15-year surv** | **20-year surv** | **25-year surv** | **P** |
| --- | --- | --- | --- | --- | --- | --- | --- | --- |
| Tobacco | HNSCC | No | 69.7% | 65.2% | 64.7% | 64.7% | 64.7% | 0.308 |
|  |  | < 20 cigarettes /day | 72.1% | 68.8% | 67.8% | 67.8% | 67.8% |  |
|  |  | ≥ 20 cigarettes /day | 68.6% | 66.4% | 66.2% | 66.2% | 66.2% |  |
|  | 2^nd^ neoplasm | No | 96.5% | 91.2% | 87.9% | 77.4% | 62.6% | 0.0001 |
|  |  | < 20 cigarettes /day | 90.5% | 79.0% | 70.6% | 58.8% | 51.7% |  |
|  |  | ≥ 20 cigarettes /day | 88.5% | 74.0% | 61.4% | 46.7% | 37.6% |  |
|  | Noncancer | No | 94.1% | 87.3% | 84.8% | 76.4% | 70.8% | 0.030 |
|  |  | < 20 cigarettes /day | 92.1% | 83.4% | 76.2% | 67.9% | 62.6% |  |
|  |  | ≥ 20 cigarettes /day | 90.9% | 84.1% | 75.9% | 67.7% | 56.8% |  |
| Alcohol | HNSCC | No | 74.2% | 70.8% | 69.8% | 69.8% | 69.8% | 0.0001 |
|  |  | <80 g alcohol/day | 75.7% | 73.2% | 73.1% | 73.1% | 73.1% |  |
|  |  | ≥80 g alcohol/day | 58.3% | 56.2% | 55.8% | 55.8% | 55.8% |  |
|  | 2^nd^ neoplasm | No | 93.7% | 85.3% | 78.8% | 69.7% | 53.6% | 0.0001 |
|  |  | <80 g alcohol/day | 89.6% | 79.1% | 69.0% | 55.5% | 46.3% |  |
|  |  | ≥80 g alcohol/day | 86.6% | 66.5% | 51.3% | 34.8% | 29.9% |  |
|  | Noncancer | No | 93.5% | 85.1% | 77.9% | 69.4% | 63.5% | 0.114 |
|  |  | <80 g alcohol/day | 91.6% | 85.3% | 78.3% | 68.6% | 60.4% |  |
|  |  | ≥80 g alcohol/day | 89.5% | 82.4% | 74.5% | 69.2% | 59.7% |  |
| Treatment | HNSCC | Surgery | 85.8% | 84.6% | 84.3% | 84.3% | 84.3% | 0.0001 |
|  |  | Surgery + (chemo)radiotherapy | 66.4% | 64.1% | 63.8% | 63.8% | 63.8% |  |
|  |  | Radiotherapy | 73.7% | 70.4% | 70.2% | 70.2% | 70.2% |  |
|  |  | Chemoradiotherapy | 65.5% | 62.7% | 60.1% | 60.1% | - |  |
|  | 2^nd^ neoplasm | Surgery | 90.7% | 82.5% | 74.2% | 59.2% | 42.1% | 0.001 |
|  |  | Surgery + (chemo)radiotherapy | 88.7% | 74.7% | 63.1% | 48.0% | 40.9% |  |
|  |  | Radiotherapy | 90.5% | 77.9% | 66.1% | 53.2% | 43.8% |  |
|  |  | Chemoradiotherapy | 88.2% | 64.8% | 55.0% | 46.6% | - |  |
|  | Noncancer | Surgery | 91.0% | 84.6% | 76.9% | 71.2% | 59.6% | 0.438 |
|  |  | Surgery + (chemo)radiotherapy | 89.6% | 83.2% | 75.8% | 64.8% | 61.0% |  |
|  |  | Radiotherapy | 91.8% | 84.1% | 76.8% | 68.8% | 58.6% |  |
|  |  | Chemoradiotherapy | 92.9% | 87.1% | 82.6% | 67.8% | - |  |
